# High-fat diet and estrogen impacts the colon and its transcriptome in a sex-dependent manner

**DOI:** 10.1038/s41598-020-73166-1

**Published:** 2020-09-30

**Authors:** L. Hases, A. Archer, R. Indukuri, M. Birgersson, C. Savva, M. Korach-André, C. Williams

**Affiliations:** 1 Science for Life Laboratory, Department of Protein Science, KTH Royal Institute of Technology, Solna, Sweden; 2 Department of Biosciences and Nutrition, Karolinska Institutet, Huddinge, Sweden; 3 Department of Medicine, Metabolism Unit and Integrated CardioMetabolic Center (ICMC), Karolinska Institutet and Karolinska University Hospital Huddinge, Stockholm, Sweden

**Keywords:** Cell biology, Risk factors, Gastrointestinal diseases, Metabolism, Nutrition, Chronic inflammation

## Abstract

There is a strong association between obesity and colorectal cancer (CRC), especially in men, whereas estrogen protects against both the metabolic syndrome and CRC. Colon is the first organ to respond to high-fat diet (HFD), and estrogen receptor beta (ERβ) can attenuate CRC development. How estrogen impacts the colon under HFD and related sex differences has, however, not been investigated. To dissect this, mice were fed control diet or HFD for 13 weeks and administered receptor-selective estrogenic ligands for the last three weeks. We recorded impact on metabolism, colon crypt proliferation, macrophage infiltration, and the colon transcriptome. We found clear sex differences in the colon transcriptome and in the impact by HFD and estrogens, including on clock genes. ERα-selective activation reduced body weight and generated systemic effects, whereas ERβ-selective activation had local effects in the colon, attenuating HFD-induced macrophage infiltration and epithelial cell proliferation. We here demonstrate how HFD and estrogens modulate the colon microenvironment in a sex- and ER-specific manner.

## Introduction

Obesity represents a worldwide public-health issue and epidemiological studies highlight a strong association between body mass index (BMI) and risk of colorectal cancer (CRC)^1–4^. A high-fat diet (HFD) increases this risk, and the incidence of CRC is increasing among young adults due to altered life-style factors^5^, but exactly how is not clear. It is known that inflammatory bowel disease (IBD) increases the risk of CRC, and that both obesity and HFD induce a general low-grade inflammatory state^6,7^. The colon is the first organ to respond to HFD^8^, with reported increase of inflammation, PPARδ signaling, stem cell activity, and altered gut microbiota^9–16^, which may all contribute to initiation of CRC. Interestingly, while obesity is a risk factor for both sexes, this association appears stronger among men^17^. Men also have a higher incidence of CRC^18,19^. A role for sex hormones is supported by epidemiological studies (reviewed in^20^), and especially estrogen appears protective against CRC^21,22^. Numerous clinical studies have also indicated that estrogen protects against several aspects of obesity and the metabolic syndrome^23,24^, as well as impacts the immune system (reviewed in^25^).

The biological action of estrogen is mediated by three receptors, the nuclear receptors ERα (ESR1) and ERβ (ESR2), and the transmembrane G protein-coupled estrogen receptor 1 (GPER1). These receptors are expressed in both sexes, with a tissue-specific expression pattern. Both ERα and ERβ have been implicated to elicit anti-obesogenic effects^26–29^. Whereas ERα clearly improves the metabolic profile, the role of ERβ in this respect is controversial^30^. Most of the CRC protective effects, however, have been linked to ERβ. Although its mRNA expression in colon is low, treatment with ERβ-selective agonist, for example, resulted in anti-inflammatory and anti-tumorigenic effects in CRC mouse models^31–34^. Furthermore, loss of ERβ results in pro-inflammatory environment and increased tumor development in both APC ^Min/+^ and colitis-associated tumor mouse model^35,36^. Notably, intestinal-specific deletion of ERβ increased tumor development and strongly enhanced inflammatory signaling in the colon of both male and female mice^37^. In fact, inflammatory signaling itself was shown to enhance transactivation of ERβ^37^. It is therefore plausible that this ERβ mechanism could protect against HFD-induced colonic inflammation. We hypothesize that estrogenic signaling, through ERα (systemic effects by improving overall metabolism), ERβ, or both; can attenuate the effect of HFD in colon, with possible sex differences.

In order to test this, and to characterize the impact of estrogen signaling on the colon microenvironment under HFD, we performed a detailed analysis of mice of both sexes. In order to compare sex differences and to obtain data comparable to premenopausal women, where obesity is thought to increase the incidence of CRC, none of the female mice in this study were ovariectomized. Mice were fed either a control diet (CD) or a HFD for 13 weeks, and administered different estrogenic ligands for the last three weeks. Importantly, by analyzing corresponding effects on the full colon transcriptome, we generated an unbiased overview of alterations mediated by HFD and estrogens. We observed sex differences in the colon transcriptome and in its response to HFD. In particular, ERβ opposed the HFD impact on colonic inflammation, epithelial cell proliferation and regulation of circadian rhythmicity. Altogether, we present the novel finding that estrogen signaling is indeed partially protective against HFD-induced alterations of the colon microenvironment, in part directly through intestinal ERβ. These findings open up new insights into prevention of obesity-associated CRC.

## Results

### Sex impacts obesity, fat distribution and fasting glucose levels

**Figure 1:**
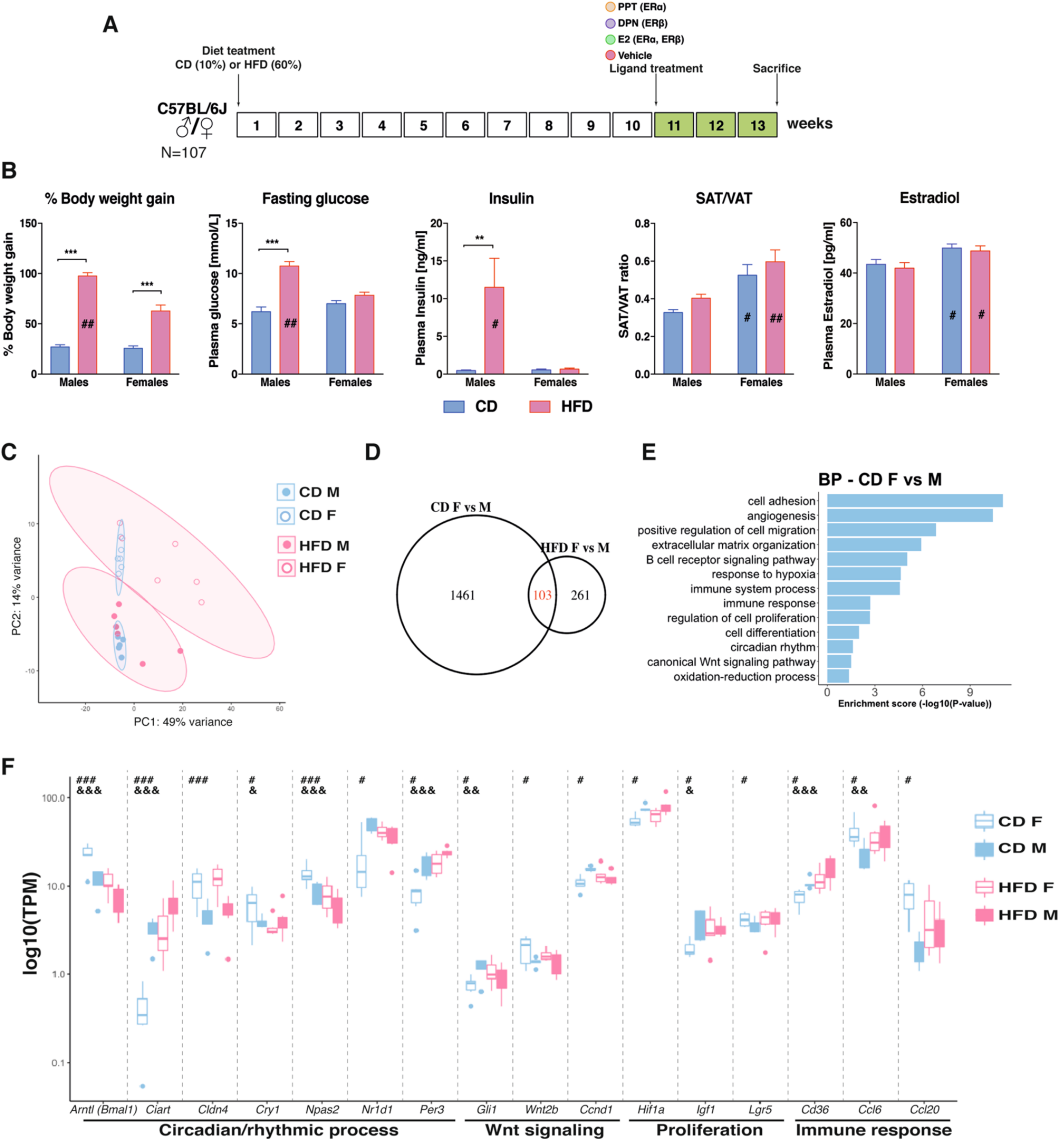
The male metabolic profile is more sensitive to HFD and the colon trancriptome exhibits sex differences independent of diet. (**A**) Experimental study design. Mice of both sexes were given CD or HFD for 13 weeks and injected with different estrogenic ligands or vehicle for the last 3 weeks prior to sacrifice. (**B**) Percentage of BW gain, fasting glucose level, insulin level, SAT/VAT ratio, and plasma estradiol levels. (**C**) Principal component analysis of RNA-seq samples for male (unfilled) and female (filled) mice fed a CD (blue) or HFD (red) (n = 5–6). (**D**) Venn diagram comparing differentially expressed genes in colon between male and female mice fed a CD or a HFD. Genes were considered as differentially expressed when *p* value < 0.05 and log_2_FC > |0.4|. (**E**) Biological process enrichment analysis based on genes differentially expressed between the sexes during CD. (**F**) Boxplot of TPM values of genes differentially expressed between sexes and/or dietary conditions. The results are presented as mean ± SEM. Two-way ANOVA followed by fisher's LSD test, *p < 0.05; **p < 0.01; ***p < 0.001. # indicate significant sex differences during CD, ## during HFD and ### during both dietary conditions; & indicate significantly difference between CD and HFD in females, && in males, and &&& in both sexes. Blue color indicates CD and pink HFD.

The impact of sex and 13-week HFD were characterized according to standard metabolic and endocrinologic measurements, including weight gain, fat distribution, fasting glucose, insulin, and circulating estrogen levels. HFD-induced weight gain was significantly higher in males compared to females (Fig. 1B). Obese males further presented significantly increased blood glucose level (after 6-h fasting) and insulin level (2-h fasting), which was not observed in obese females (Fig. 1B). These results support previous observations that obese males are more susceptible to glucose intolerance and insulin resistance compared to females^38,39^. As also noted previously^39^, the fat distribution was sex-dependent. Males had more visceral white adipose tissue (VAT) and less subcutaneous white adipose tissue (SAT) in relation to total fat during both dietary conditions, compared to females (Supplementary Fig. 1A). As a consequence, males had a substantially lower SAT/VAT ratio (Fig. 1B). A low SAT/VAT ratio has been correlated to poor metabolic outcomes. As expected, female mice had significantly higher circulating estradiol levels compared to males during both CD and HFD (Fig. 1B). Circulating estradiol levels were not significantly impacted by obesity (Fig. 1B). This contradicts one previous study which found increasing circulating estradiol levels upon HFD^30^. However, obesity is primarily known to increase local levels of estrogen in adipose tissue, which express aromatase^40^. We did not measure local adipose tissue estradiol levels. Overall, these results and sex differences replicate previous findings.

### The colon transcriptome exhibits clear sex differences

To specifically study sex differences in the colon, we performed RNA-seq of colon tissue from males and females. As evident from the principal component analysis (PCA) plot, the colon transcriptomes exhibited clear sex differences, especially during CD (Fig. 1C). Gene expression analysis also identified 1564 significantly differentially expressed genes (DEG) between male and female colon under CD (Fig. 1D). While the CD colon transcriptome clustered tightly together, the HFD was more spread out (Fig. 1C). As a result, the sex difference under HFD was less distinct, but 364 DEGs were still revealed (Fig. 1D). For animals fed a CD, enrichment analysis for biological processes (BP) disclosed that the genes responsible for the sex differences were related to immune response (e.g. *Cd36, Ccl6* and *Ccl20*), cell proliferation (e.g. *Hif1a, Igf1* and *Lgr5*) and canonical Wnt signaling (e.g. *Ccnd1, Gli1* and *Wnt2b,* Fig. 1E,F). Although the mice had been sacrificed at the same time of the day, we found sex differences in expression of circadian genes, including the clock genes *Arntl (Bmal1), Npas2*, *Cry1, Per3,* and *Nr1d1/Rev-ErbA* (Fig. 1E,F). Over one fourth (28%) of the sex differences after HFD were also present under CD (Fig. 1D). The most overrepresented genes among these stable DEGs (103) belonged to the circadian/rhythmic pathway (e.g. *Bmal1, Ciart, Cldn4* and *Npas2,* Fig. 1F). Thus, we report for the first time that mice fed CD, which lacks phytoestrogens, display clear sex differences in the colon transcriptome.

### HFD modulates the colon transcriptome differently in males and females

**Figure 2:**
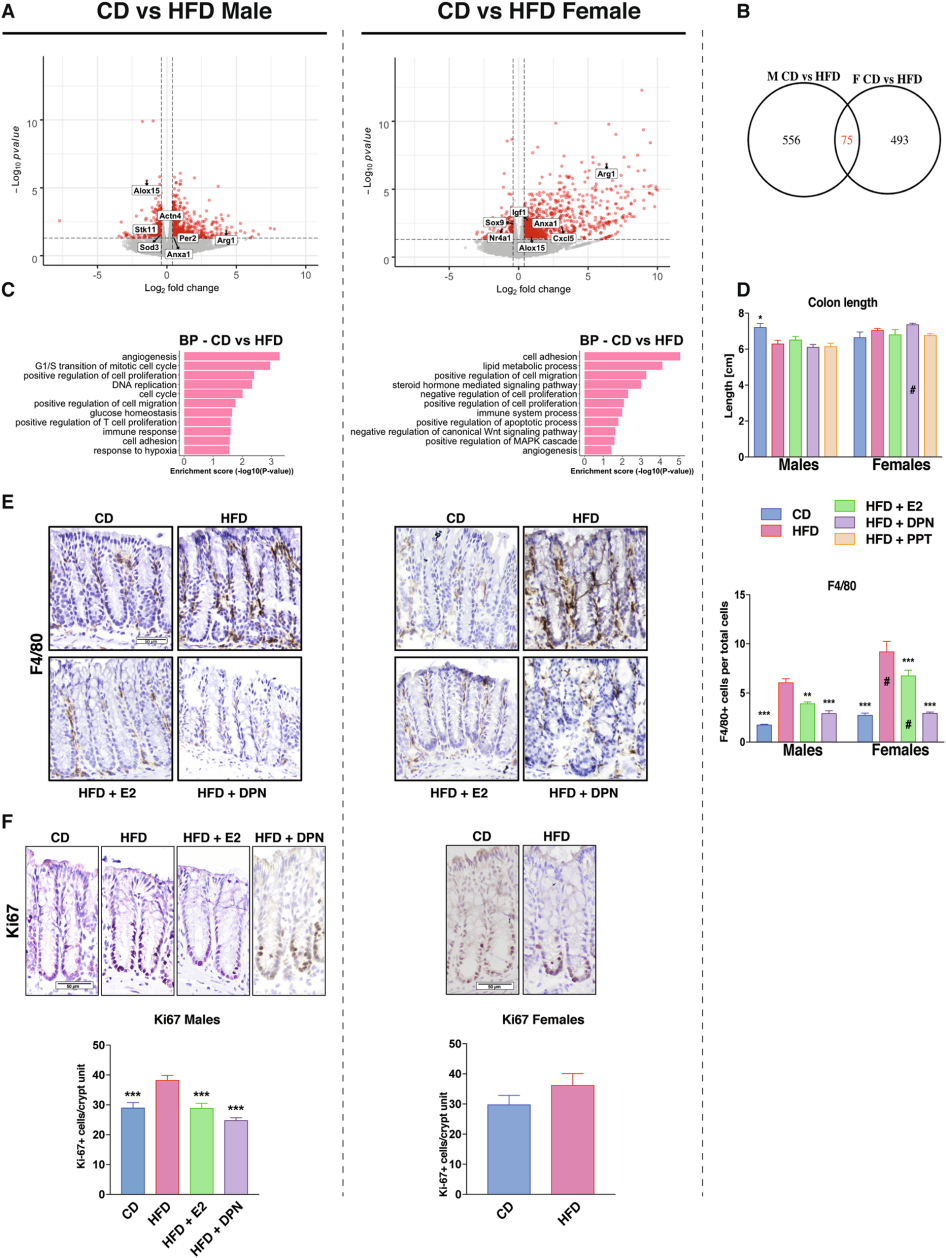
The colon transcriptome responds to HFD in a sex-dependent manner. (**A**) Volcano plots show HFD-regulated genes in males (left) and females (right). Genes were considered differentially expressed when *p* < 0.05 and log_2_FC > |0.4|, indicated in red. (**B**) Venn diagram comparing differentially expressed genes in colon between CD and HFD in male and female mice. (**C**) Biological process enrichment analysis of HFD-regulated genes, separated by sex. (**D**) Colon length measured in cm. (**E**) Macrophage infiltration measured by F4/80^+^ IHC and quantified by stained cells per total cells ratio. (**F**) Intestinal epithelial cell proliferation measured with Ki67 IHC and quantified by number of positive cells per crypt unit. n = 5–15 per group. Results are presented as mean ± SEM. One-way and two-way ANOVA with uncorrected Fisher’s LSD test. *Indicate significant differences compared to HFD + vehicle, *p < 0.05; **p < 0.01; ***p < 0.001. ^#^Indicate sex differences.

In order to explore how HFD impacted male and female colon, we compared the transcriptomes under HFD with corresponding expression under CD, for males and females separately. We identified 568 HFD-modulated DEGs in females and 631 in males (Fig. 2A). We noted that a larger proportion of genes was upregulated overall, but especially in females (85%, versus 51% in males), and that females exhibited stronger upregulation. Most of the modified genes differed between the sexes, only 75 genes responded to HFD in both (Fig. 2B). Among these common genes were, again, those related to the circadian rhythm (*Bmal1, Ciart, Npas2* and *Per3,* Fig. 1F), oxidation–reduction process (*Alox15*), and inflammatory response (e.g. *Arg1* and *Anxa1,* Fig. 2A). Both sexes altered expression of genes with functions in cell adhesion, cell proliferation, angiogenesis, migration, and immune system (Fig. 2C and Supplementary Table 2), but different genes were altered in males and females. Regulated genes involved in cell cycle (e.g. *Stk11* and *Wee1*), hypoxia (e.g. *Actn4* and *Sod3*) and glucose homeostasis (e.g. *Stk11*) were particularly enriched in males, whereas regulation of apoptosis (e.g. *Nr4a1*), inflammatory response (e.g. *Cxcl5*), MAPK cascade (e.g. *Igf1*) and Wnt signaling (e.g. *Sox9*) were enriched in females (Fig. 2A,C and Supplementary Table 2). Thus, while both sexes presented alterations within similar biological functions, including the immune system and cell adhesion, other changes appeared tied to sex, such as more pronounced changes of cell cycle and hypoxia in males, and of the lipid metabolism, steroid hormone and Wnt signaling in females. All together our data demonstrate, for the first time, that sex impacts the gene expression response to HFD in colon.

### HFD increases colon epithelial inflammation and proliferation in males

The above colon transcriptome analysis indicated that the immune system was impacted by HFD and that, especially in males, the cell cycle and hypoxia were modulated. A shortened colon is indicative of colonic inflammation, and we found that males but not females fed an HFD presented a significantly shorter colon (Fig. 2D). Next, we explored markers for macrophages in histological sections using immunohistochemistry (IHC). We found that mice of both sexes exhibited a significantly increased F4/80^+^ macrophage infiltration when fed HFD, but females had a stronger infiltration than males (Fig. 2E). Males on the other hand, presented significantly increased proliferation of crypt cells after HFD, which females did not (Fig. 2F). This reveals that HFD results in clear sex-dependent functional defects in the colon, in accordance with the above noted transcriptional differences.

### Estrogen improves the metabolic profile of obese males via ERα

**Figure 3:**
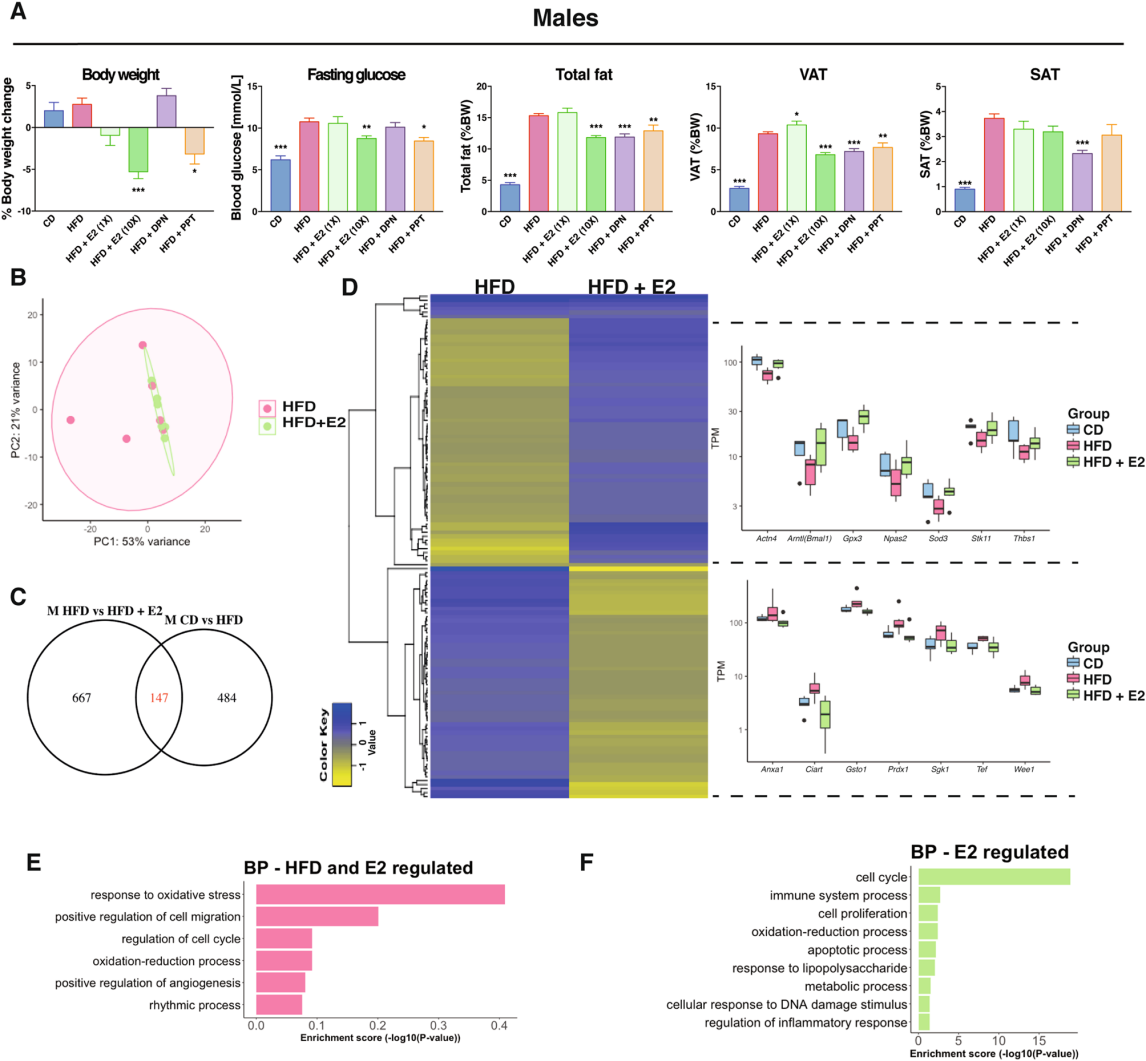
Estrogen treatment impacts the metabolic profile and colon gene expression in males. (**A**) BW change during treatment, fasting glucose level, total fat mass (reported to BW), VAT, and SAT (reported to BW) for males on a CD, HFD, and HFD treated with E2, DPN and PPT. (**B**) PCA of RNA-seq data for males fed a HFD with and without E2 treatment. (**C**) Venn diagram comparing HFD-regulated genes in males with E2-regulated genes in males under a HFD. (**D**) Heatmap of the homogenously regulated common genes (regulated by both HFD and E2 under HFD) and boxplots of TPM values of some genes. Biological process enrichment analysis of the (**E**) HFD-induced genes opposed by estrogen signaling and (**F**) estrogen-regulated genes under HFD feeding (n = 5–15 per group). Results are presented as mean ± SEM. One-way ANOVA with uncorrected Fisher’s LSD test. * Indicate significant differences compared to HFD + vehicle, *p < 0.05; **p < 0.01; ***p < 0.001.

Several studies have shown that estradiol (E2) improves the metabolic profile of HFD-fed rodents of both sexes^41,42^. Further, knockout of ERα results in obese mice^26^. In our study, neither E2 nor ERα-selective ligand PPT treatment for the last 3 weeks (Fig. 1A) affected the body weight (BW)-gain in females compared to vehicle treatment (Supplementary Fig. 1C,D). However, male mice showed a dose-dependent BW loss during E2 treatment, with up to 5% weight reduction (0.5 mg/kg BW, Fig. 3A and Supplementary Fig. 1C). This was accompanied by a significant reduction of total fat and decreased blood glucose level upon fasting, not seen in females (Fig. 3A and Supplementary Fig. 1D). These effects were also evident with the selective ERα ligand PPT (Fig. 3A). Thus, our experimental set-up confirms that exogenous E2 via ERα impacts obesity, fasting glucose, total fat and VAT ratios in males.

### Estrogen impacts the obese colon transcriptome

Next, we wanted to determine if E2 treatment impacted the colon, and specifically if it could modulate the HFD-induced colon inflammation signature. We focused on males, who had exhibited a shortening of the colon upon HFD and had shown a significant E2-mediated impact on their metabolic profile. The RNA-seq data, visualized in a PCA plot, showed that estrogen-treated HFD-fed male colon clustered tighter together compared to vehicle treated (Fig. 3B), just as CD samples did (Fig. 1C). We found that E2 treatment modified the expression of 814 genes in distal colon. Among these E2-regulated genes, we noted enrichment of cell cycle and cell proliferation (e.g. *Cdk1*, *Melk*, *Mki67*), and immune system process (e.g. *Alcam*, *Irf7* and *Bcl6*) pathways (Fig. 3F). Of these, 147 genes were also impacted by HFD (compared to CD), nearly all (97%) in the opposite direction (Fig. 3C,D). BP enrichment analysis revealed that genes regulated by both HFD and E2 were involved in cell cycle and rhythmic processes (Fig. 3E). Two homogenous (regulated similarly in at least three animals from each group) clusters of DEG appeared, illustrated by a heatmap in Fig. 3D. One cluster (61 genes) was downregulated by HFD and then upregulated by E2, such as *Actn4, Bmal1*, *Gpx3, Npas2*, *Sod3, Stk11* and *Thbs1* (Fig. 3D, upper right). The second cluster included 58 genes that were upregulated by HFD and downregulated by E2, and comprised genes such as *Anxa1*, *Ciart*, *Gsto1*, *Prdx1*, *Sgk1, Tef* and *Wee1* (Fig. 3D, lower right). qPCR supported these regulations. This demonstrates that both sex and estrogen have a substantial impact on the colon transcriptome, that estrogen may have a strong effect on proliferation and clearly opposes a fraction of the HFD-induced expression.

### Estrogen reduces immune cell recruitment and proliferation of the HFD colon

Since we noted that E2 treatment could attenuate signs of inflammation and proliferation at the gene expression level, we investigated whether E2 impacted the length of colon, infiltration of macrophages, or proliferation. E2 did not significantly impact the colon length but did reduce F4/80^+^ macrophage infiltration in both sexes (Fig. 2D,E). We also noted a significant reduction of positive Ki67-stained cells upon E2 treatment of HFD males (Fig. 2F). These data clearly demonstrate that E2 impacts the colon, both in terms of immune cell recruitment and colonic epithelial cell proliferation.

### ERβ-selective agonist impacts fat distribution and reduces colonic macrophages infiltration and cell proliferation

To investigate ERβ-mediated effects, we included treatment with a ligand selective for ERβ (DPN, Fig. 1A). DPN treatment did not result in weight reduction, in accordance with data assigning this phenotype to ERα. On the contrary, DPN-treated males and females exhibited a non-significant trend of increased weight gain, which was accompanied by a significant increase of fasting blood glucose levels in females but not males (Fig. 3A and Supplementary Fig. 1D). This also demonstrates that the ligands in the concentrations used in this set up retained receptor selectivity. Although the effects by DPN on weight were not significant in males, we noted clear reductions of total fat and VAT by DPN treatment, in a scale similar or higher than what was achieved with PPT treatment (Fig. 3A). Further, a reduction in SAT was specific for ERβ ligand activation (Fig. 3A). We did not see these effects in females where, on the contrary, VAT was increased by DPN (Supplementary Fig. 1D). This suggests that while obesity and HFD-induced BW can be decreased only through ERα, ERβ activation appears to modify the ratio of total fat, VAT, and SAT in males, and increase fasting glucose and VAT in females. Furthermore, estrogen through ERβ (E2 and DPN) opposed the HFD-induced Ki67 proliferation in males (Fig. 2F). The increased F4/80^+^ macrophage infiltration upon HFD was also significantly reduced by estrogen through ERβ (E2 and DPN) in both sexes (Fig. 2E). Our data thus establish that while an improved metabolic profile is mediated via ERα, ERβ impacts the colon by attenuating HFD-induced colonic proliferation in males and macrophage infiltration in both sexes.

### ERβ regulates gene expression in the colon

**Figure 4:**
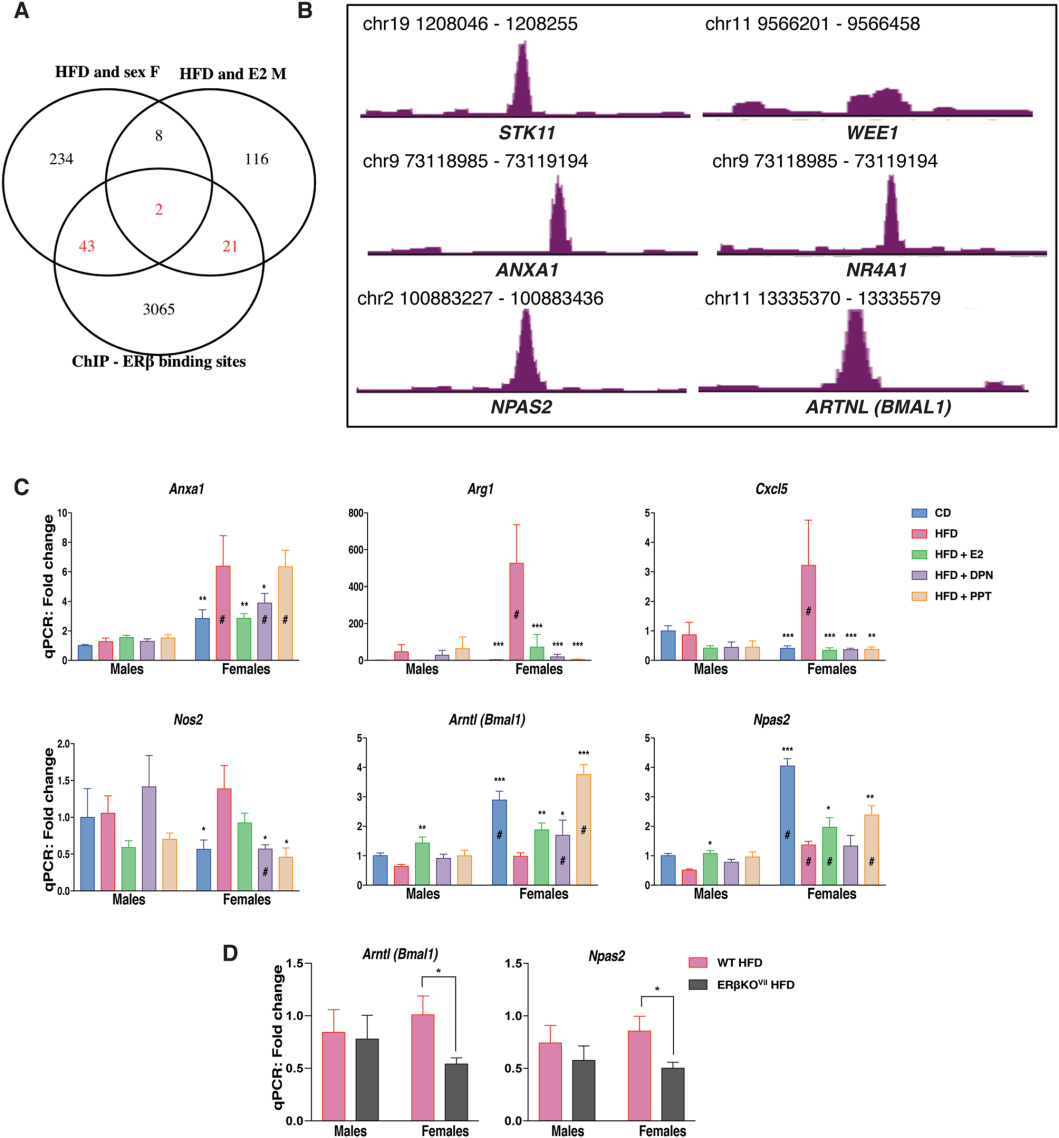
Estrogen through both ERα and ERβ can modulate the expression of specific sets of genes under HFD in both sexes. (**A**) Venn diagram of the E2-regulated genes under HFD in males and the HFD and sex regulated genes in females compared to ERβ-binding chromatin sites in two different CRC cell lines (SW480 and HT29). (**B**) ERβ-binding sites revealed by ChIP-seq in CRC cell lines. (**C**) qPCR measurement of gene expression in the colon of female and male mice on a CD, HFD and HFD treated with E2, DPN and PPT (n = 5–15). (**D**) qPCR measurement of gene expression in colon of wild type and intestinal-specific ERβ KO mice fed a HFD. The results are presented as mean ± SEM. Two-way ANOVA with uncorrected Fisher’s LSD test. * Indicate significant differences compared to HFD + vehicle, *p < 0.05; **p < 0.01; ***p < 0.001. ^#^Indicate sex differences.

As shown above, E2 treatment opposed a set of HFD-induced colonic gene expression in males. This could be an indirect consequence of systemic effects (e.g. ERα improving the overall metabolism) or direct local effects through ERs expressed in the colon. We have previously shown that ERβ is expressed in intestinal epithelial at low levels, whereas there is not sufficient support that ERα is expressed in the colon^43^. While we did not sequence the colon transcriptome of DPN-treated mice, we evaluated the E2-mediated response of ERβ-target genes (as predicted from our ERβ chromatin immunoprecipitation (ChIP)-seq data from human CRC cell lines, with transduced ERβ, GSE149979) in the male colon transcriptome. This resulted in identification of 23 genes, which were regulated by both HFD and E2, and also have cis-chromatin ERβ-binding sites (Fig. 4A). These included the cell cycle genes *Stk11* and *Wee1* and the inflammatory related gene *Anxa1* among others (Fig. 4B). Moreover, we compared the female-only HFD-regulated genes (potential female endogenous E2 regulation). This comprised of 287 genes of which 45 had ERβ-binding sites, including the orphan nuclear receptor *Nr4a1 (Nur77,* Fig. 4A,B). qPCR analysis of colon tissue corroborated the HFD-induced upregulation of *Anxa1* gene expression and that this was indeed attenuated by both E2 and DPN (ERβ) but not by PPT in females, whereas it was not affected in males (Fig. 4C). Further, the HFD-induction of the M2 macrophage marker *Arg1* was also stronger in females, and this was blocked by both ERα and ERβ activation (Fig. 4C). In addition, we have previously shown that ERβ can cross-talk with and regulate NFκB signaling in both human colon cell lines and mouse in vivo colon (ERβ-intestinal knockout mice)^37^. In line with this, we here observed that HFD-induced expression of several NFκB target genes (*Cxcl5, Nos2)* in females, which were blocked by both ERβ (DPN) and ERα (PPT). Our data suggest that systemic activation of ERα improves the overall metabolism, which helps oppose obesity-mediated colon dysfunction. ERβ, on the other hand, appears to locally downregulate proliferation and inflammatory genes (*Cxcl5* and *Nos2*) in the colon. This data provides evidence that E2 through systemic and local effects via ERα and ERβ can partially attenuate inflammatory effects in the colon.

### Sex, hormones, and diet influence regulation of circadian clock genes in colon

The circadian rhythm is essential for intestinal homeostasis. Disruptions can increase intestinal permeability, proliferation, exacerbate colitis, modulate microbiota composition, and impact the immune system^44–47^. HFD impacts the circadian rhythmicity in both liver and adipose tissue, but very little is known about the impact of HFD on the circadian rhythm in the colon epithelium. Above, we identified sex differences in the expression of clock genes with higher expression of *Bmal1, Npas2,* and *Cry1* in female colon and higher expression of *Per3* and *Nr1d1 (Rev-ErbA)* in male colon (Fig. 1F). HFD downregulated *Bmal1* and *Npas2* and upregulated *Per3* in both sexes, downregulated *Cry1* specifically in females and upregulated *Per2* specifically in males (Fig. 1F, 2A). In addition to exhibiting sex differences in their expression levels, ER ligands could oppose the HFD-induced downregulation of both *Bmal1* and *Npas2* in both sexes (Fig. 3D, 4C). ChIP-seq indicated that ERβ is a regulator of the clock genes *Bmal1* and *Npas2* (Fig. 4B). In males, both *Bmal1* and *Npas2* were upregulated in the colon by estrogen, but significant regulation by ERβ (DPN) were not observed (Fig. 4D). However, we could confirm that the circadian clock gene *Bmal1* indeed was upregulated by DPN (ERβ) and, also, by E2 and PPT (ERα) in females (Fig. 4B). This evidence points towards local regulation of clock genes by ERβ in the colon. The circadian rhythm of the colon has essential impacts on the body metabolism. If this is impacted by ERβ, as our study shows, this has substantial implications for our understanding of how hormones affect the metabolism. To provide conclusive evidence of direct ERβ-mediated regulation of *Bmal1* and *Npas2* in the in vivo female colon, we fed intestinal-specific ERβ knockout mice HFD. We observed a significant decrease in the expression of both *Bmal1* and *Npas2* in female colon lacking intestinal ERβ (Fig. 4D). This was not observed in males. Our results demonstrate, that the expression of genes in colon, including clock genes, has clear sex differences, is impacted by HFD in both sexes, and is directly regulated by intestinal epithelial ERβ in the colon of females.

## Discussion

Our objective with this study was to determine whether estrogen could improve the colon microenvironment and thereby attenuate the negative effects of HFD and, if so, to dissect this mechanism and identify sex differences. Several studies have suggested that both ERα and ERβ can improve the metabolic syndrome, a risk factor for CRC^26–28^. Other studies support a protective role of ERβ against CRC (reviewed in^19^). The role of ERβ, or ERα, in the colon during HFD-induced obesity has, however, not been investigated. We here demonstrate that estrogen indeed modulates the colon microenvironment in mice fed HFD, and that ERα- and ERβ-selective ligands can in part counteract different facets of HFD-induced phenotypes, including at the level of colonic gene expression.

As the metabolic data show, males appeared more sensitive to HFD, and the higher-dose E2 and ERα-selective activation improved their glucose metabolism, in line with previous studies^38,41^. Although both doses of E2 injections resulted in similar plasma concentrations, the low-dose E2 did not significantly impact glucose metabolism. It is possible that the higher dose resulted in increased local concentrations in certain tissues, especially as lipophilic steroid hormone can be sequestered in the visceral adipose tissue, or that it was higher at certain time points, not reflected in the plasma concentrations at the point of sacrifice. Some studies have also reported a protective effect through ERβ against HFD-induced obesity, but the data are conflicting. Foryst-Ludwig et al. found that ERβ protected against HFD-induced weight gain but still elicited pro-diabetogenic effects^30^. We could not confirm that ERβ protected against HFD-induced weight gain, but we did find that its activation (E2 and DPN, not PPT) impaired glucose levels in females (Supplementary Fig. 1D).

Our study detail several other significant sex differences. In particular, a notable sex difference in the colon transcriptome of mice (CD), which was not expected. As this diet did not contain phytoestrogens, which standardized soy-based chow diets are rich in, this enabled us to observe the impact of endogenous estrogen signaling without external interference. The sex differences that were observed in both dietary conditions (CD and HFD) were related to hypoxia, immune response, cell proliferation, circadian rhythm and Wnt signaling. Thus, our data bring in a role of sex hormones in the basal regulation of these pathways in colon. Intriguingly, we found that estrogen treatment in males indeed regulated the immune response, cell proliferation and rhythmic processes in colonic tissue.

Interestingly, HFD-induced pro-inflammatory modifications are known to occur earlier in the colon than in the adipose tissue. These changes are characterized by increased infiltration of pro-inflammatory macrophages and by epithelia permeability in the colon^8^. Estrogen treatment has been shown to elicit an anti-inflammatory response in the colon of mouse models for colitis^32,48^. Our transcriptomic data reveal that both sexes' immune response were altered upon HFD feeding, but with clear sex differences. This was supported by a significant increase of F4/80^+^ macrophage infiltration in the colon upon HFD in both sexes, but with a significantly higher overall macrophage count in females. The F4/80^+^ macrophage infiltration was significantly reduced by ERβ activation (E2 and DPN) in both males and females. In addition, our mechanistic data from ChIP-seq in CRC cell lines demonstrated that ERβ could bind to cis-regulatory chromatin regions of both *Anxa1* and the orphan nuclear receptor *Nr4a1 (Nur77)*. *Anxa1* is known to recruit monocytes^49^ and *Nur77* can regulate the immune system and suppress DSS-induced colitis^50^. In females, we found that ERβ activation could reduce the expression of *Anxa1* and markers for anti-inflammatory M2 (*Arg1*) and pro-inflammatory M1 (*Nos2*) macrophages. Thus, our study delineates how ERβ contributes to anti-inflammatory effects mediated by estrogen in the in vivo colon. ERβ is not detected in macrophages, but is expressed in intestinal epithelial cells^51,52^. Cross-talk between the colonic epithelial cells and macrophages has been demonstrated in IBD^53^ and with HFD in an obesity context^8^. Although the etiology of obesity and IBD is different, both are characterized by gut inflammation with common inflammatory pathways including TNFα, IL6, and IL1b^54–57^. We have recently shown in a colitis-induced CRC mouse model that ERβ in intestinal epithelial cells can downregulate the TNFα/NFkB signaling^37^. Together, our data suggest that specific activation of intestinal ERβ may have a beneficial impact to modulate gut inflammation in both pathologies.

Obesity is a well-known risk factor for CRC, and HFD-induced obesity exacerbates colon tumor development in mice by enhancing colonic cell proliferation^58^. We reveal that the colon transcriptome, which responded to HFD-feeding in a sex-dependent manner, were particularly enriched for pathways involved in cell proliferation. HFD clearly increased and E2 treatment opposed the HFD-induced proliferation, both at the transcriptome and functional (Ki67) level, in males. While we saw a trend of HFD-increased proliferation in females, this was not significant and we assume that they may be protected through their already higher levels of endogenous estrogen. Previous in vitro results have demonstrated anti-proliferative effects by exogenously expressed ERβ in CRC cell lines^59–61^. In line with these data, we found a significant decrease in HFD-induced cell proliferation upon ERβ activation (DPN). These results demonstrate that ERβ can protect against the HFD-induced cell proliferation in males, and potentially undermine the promotion of colon tumor development.

The most unexpected finding was that key circadian clock genes were differentially expressed between the sexes in the colon, during both dietary conditions. The circadian clock regulates critical processes involved in inflammation and proliferation. The circadian rhythm has been shown to be dysregulated in both obesity and IBD^62,63^. HFD feeding is known to impact the circadian rhythm in the hypothalamus, liver and fat tissue, and to disrupt the eating behavior. However, timed HFD-feeding can reset the circadian metabolism and help prevent obesity and metabolic disorders^64,65^, indicating a major contribution of the circadian rhythm in the pathogenesis of the disease. The core circadian clock is regulated by different factors including sex hormones such as estrogen and there are evidences of sex-dependent regulation^66^. In breast cancer cell lines, ERα was shown to increase the protein and mRNA expression of Clock^67^, indicating the potential of estrogen receptors to regulate clock genes at the tissue level. However, the impact of HFD and estrogen on the circadian rhythm in the colon epithelium has not been investigated previously. Here we show that mice fed HFD, of both sexes, alter expression of key clock genes *Npas2* and *Bmal1*, and that estrogen can modulate this expression. Estrogen is also known to impact the feeding behavior^68^. The food intake was not successfully monitored in this study, due to problems with the HFD breaking into powder, and we cannot exclude the possibility that some of the effects mediated by estrogen could be through decreased diet consumption. However, our mechanistic data demonstrated that ERβ could bind to cis-regulatory chromatin regions of both *Npas2* and *Bmal1*. This suggests that ERβ acts locally in the colon and directly regulates these genes, and we could confirm this using our intestinal-specific ERβ knockout mice. The expression of colonic *Bmal1*, which was enhanced by selective-ERβ activation in females, was indeed significantly decreased upon colon epithelia ERβ knockout in female mice on a HFD. It is worth noting that ERα activation by PPT also regulated the expression of *Npas2* and *Bmal1* in the colon*.* Available data do not support significant expression of ERα in the colon^51^, and we therefore assume that these effects are systemic through its effect on the overall metabolism. However, we did notice a two-fold increase of colonic ERα mRNA levels in female colon upon HFD-feeding (data not shown), and we cannot completely exclude local effects of ERα in colon, possibly via immune cells. The ERβ-binding chromatin site by *Bmal1* included an estrogen response element (ERE), which ERα should also be able to bind, if expressed. However, the *Npas2* ERβ-binding site was indicated to be through tethering with JUN/AP-1. Since the two ERs differ in their protein interactions, because of less conserved AF1 and AF2 domains, this site is more likely to be specific for ERβ. It should be emphasized that although all mice were sacrificed at the same time of the day, this was not a circadian experiment, and we also did not monitor the estrous cycle of the females. Hence, while our results highlight the implication of ERβ in the regulation of core clock genes in the colon, further experiments investigating different zeitgeber times are needed to conclude its impact on their rhythmicity. Additionally, further studies are needed to investigate the impact of different estrous phases on the colon transcriptome, since our results in females presents the average response of diet, independent of the estrous cycle.

**Figure 5:**
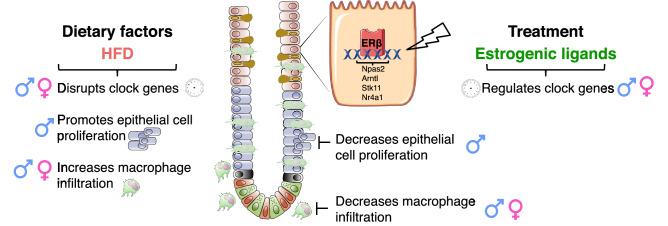
Schematic illustration; proposed model for estrogen regulation of the colon microenvironment during HFD-induced obesity. The HFD-induced impaired colonic circadian clock genes and increased cell proliferation was opposed by ERβ in males. The HFD-induced increase in macrophage infiltration was repressed by ERβ activation in both females and males.

In conclusion, our data provide evidence that the colon microenvironment responds to HFD in both sexes, but with apparent sex differences, as illustrated in Fig. 5. Both sexes present an altered expression of core clock genes, although this was stronger in females, and increased macrophage infiltration. Males, on the other hand, showed significant effects on epithelial cell proliferation, increased by HFD and decreased through ERβ. All of the above functions play important roles in the pathogenesis of HFD-induced metabolic disorders and colitis. Entirely novel findings in our study demonstrate that estrogen signaling can partially modulate the colon microenvironment during HFD, and that core clock genes*,* colonic cell proliferation and macrophage infiltration can be modulated by estrogen via ERβ in this context, in a sex-dependent manner. Intestinal-specific ERβ knockout mice and ChIP-seq data demonstrate that ERβ in the colon is a regulator of both *Bmal1* and *Npas2*. This study opens up new insights, where ERβ exhibits beneficial action against deleterious effects of diet-induced obesity on colon microenvironment.

## Methods

### Animal experiment and tissue collection

Five to six-week old male and non-ovariectomized female C57BL/6J mice were obtained from in-house breeding. Mice lacking ERβ specifically in the epithelial cells in the intestine were generated by crossing ERβ^flox/flox^ mice (B6.129X1- Esr2^tm1Gust^, Jan-Åke Gustafsson’s laboratory) with the transgenic mice bearing Cre recombinase expressed under the control of the enterocyte-specific Villin promoter (B6.SJL-Tg(Vil-cre)997 Gum/J; Jackson Laboratory, Bar Harbor, ME). Littermates (ERβ^flox/flox^) lacking the Cre allele were used as controls (referred to as WT). The genotype was confirmed with standard PCR protocol. Animals were housed under controlled environment at 20 °C with a 12-h light–dark cycle. The animals were fed HFD (D12492, 60% kcal fat, Research Diet) or a control diet corresponding to a matched low-fat diet (D12450J, 10% kcal fat, Research Diet) and water was provided ad libitum. For the treatment with estrogenic ligands, at 10 weeks of diet the mice were injected *i.p.* every other day for a total of 9 injections with 0.05^69^ (N = 10 for males and 11 for females) or 0.5 mg/kg^70^ (N = 10 for males) BW for 17β-estradiol (E2, Sigma-Aldrich ), 2.5 mg/kg BW for 4,4′,4′'-(4-Propyl-[1H]-pyrazole-1,3,5-triyl)trisphenol (PPT, Tocris, N = 5 per group for both females and males)^71^, 5 mg/kg BW for 2,3-*bis*(4-Hydroxyphenyl)-propionitrile (DPN, Tocris, N = 10 for males and 9 for females)^71^, or vehicle. The ligands were prepared in a solution of 40% PEG400, 5% DMSO and 55% water. Mice were fasted for 6 h prior the measurement of the glucose level. Briefly, a small piece of the tail was cut with a scalpel, a drop of blood was removed, and glucose level immediately measured with a OneTouch Ultra glucometer (AccuChek Sensor, Roche Diagnostics). Mice were dissected and fat pads from the abdominal and posterior subcutaneous regions carefully removed and weighted. Total fat (TF) was calculated as the sum of all fat depots including gonadal fat, retroperitoneal fat, omental fat and inguinal/gluteal fat depots. Visceral adipose (VAT) comprised of the sum of gonadal fat, retroperitoneal fat and omental fat. The subcutaneous fat (SAT) comprised of the inguinal/gluteal fat depots. All experiments were performed in accordance with ARRIVAL guidelines and EU Directive 2010/63/EU, for the care and use of laboratory animals. The local ethical committee of the Swedish National Board of Animal Research (Stockholm ethical committee, N230/15) approved all experimental protocols. Colons were harvested, washed, measured and opened along the long axis. The colons were then either embedded in OCT (Tissue-tek, Sakura) and prepared for IHC analyses using the “swiss roll” method, or snap frozen in liquid nitrogen for further analysis.

### ELISA

Blood was collected by cardiac puncture at sacrifice using EDTA-treated tubes and plasma was collected after centrifugation and stored at -80 for further analysis. Estradiol levels were measured in mouse plasma by mouse/rat estradiol kit (ES180S-100, Calbiotech) according to manufacturer’s instructions. Plasma samples were diluted 1:5 for vehicle treated CD and HFD and 1:12 for E2 treated mice with assay diluent. Insulin level was measured in plasma by mouse/rat Elisa kit (EZRMI-13K) according to manufacturer’s instructions with plasma samples dilution of 1:2. Calculations were performed using standard controls provided with the kit.

### IHC

Colonic samples embedded in OCT were cryo-sectioned at 8 μm thickness. Sections were fixed in 4% formaldehyde for 10 min and endogenous peroxidase activity was blocked with 3% hydrogen peroxidase in 50% methanol and PBS for 30 min. Slides were blocked with 5% normal goat serum for 30 min at 4 °C and blocked for unspecific avidin/biotin binding (DAKO). The sections were incubated overnight at 4 °C with the primary antibodies, anti-F4/80^+^ (1:100, cat# MCA497R, lot# 1608, RRID: AB_323279, Bio-Rad), anti-Ki67 (1:100, SP6, cat# MA5-14520, lot# TC2542944A, RRID AB_10979488, Invitrogen) in 0.1% IGEPAL in PBS. Negative controls without primary antibodies were used for each slide. The sections were incubated with appropriate biotin conjugated anti IgG secondary antibodies (1:500) for 1 h at room temperature (RT), and then incubated with avidin–biotin complex (ABC, Thermofisher) for 1 h at RT. Sections were developed with Liquid DAB + (3,3-diaminobenzidine) Substrate Chromogen System (DAKO) and counterstained with Mayer’s Hematoxylin (Sigma-Aldrich). After dehydration in ethanol and xylene, the slides were cover-slipped using Pertex (Histolab). Ten to fifteen random images from three fully stained sections per animal were randomly taken using a BX53 light microscope and CAM-SC50 camera (Olympus). Positive stained cells per crypts were counted using QuPath and approximately 30 crypts per animal were analyzed.

### RNA isolation and qPCR

Frozen colon distal tissue and colon epithelial layer were homogenized with a tissue lyser (Qiagen, Chatsworth, CA). Total RNA was isolated with QIAzol and purified using miRNeasy Mini Kit (Qiagen, Chatsworth, CA) according to the standard protocol and on-column DNAse treatment was applied. Quantitative and qualitative analyses of the RNA were performed with NanoDrop 1000 spectrophotometer and Agilent Tapestation 2200 (Agilent Technologies, Palo Alto, CA), respectively. All samples had RNA integrity > 6.5. One microgram RNA was reversed transcribed using iScript cDNA synthesis kit (Biorad) according to standard protocol. Ten nanogram of cDNA were used to perform qPCR in the CFX96 Touch System (Biorad), with iTaq universal SYBR Green supermix (Biorad) as recommended by the supplier. Samples were run in duplicates and the relative gene expression was calculated as the mean per group using the ΔΔCt method, normalized to the geometric mean of three reference genes (*Actb*, *Eef2* and *Tbp*). Primer sequences are provided in supplementary table 1.

### RNA-seq

RNA from colon tissue was prepared from 6 biological replicates, from male and female fed with CD and HFD, and males fed with HFD and treated with E2 (0.5 mg/kg BW). Library preparation (Illumina RiboZero) and sequencing (Illumina NovaSeq6000) was performed at Sweden’s National Genomics Infrastructure (NGI). At least 17 M paired-end reads (2 × 51 bp in length) were generated for each sample, and mapped against mouse genome (GRCm38) using STAR. FeatureCounts and StringTie were used to generate gene counts and TPM values. DESeq2 was used to calculate differentially expressed genes (DEG) with raw counts as input and the Benjamini–Hochberg procedure was used to estimate FDR. Genes were considered as significantly differentially expressed if *p* value < 0.05 and log2FC >|0.4|. Gene ontology/biological function was performed with DAVID bioinformatics website.

### Statistical analysis

GraphPad Prism was used for statistical analysis (GraphPad Software Inc, La Jolla, CA). The results are presented as mean ± SEM. A (two-tailed) Welch's t-test was used for comparison between two groups. One-way analysis of variance (ANOVA) was used for comparison between multiple conditions followed by fisher's LSD test. Two-way ANOVA was used for comparison between multiple conditions in two different groups followed by fisher's LSD test. All conditions were compared to HFD vehicle and a *p* value < 0.05 was considered being statistically significant (*p < 0.05, **p < 0.01, ***p < 0.001).

## Supplementary information

Supplementary information.

## Data Availability

Gene expression data are deposited in the NCBI Gene Expression Omnibus database [GSE149811].
