# Supplementary material for: High-fat diet and estrogen impacts the colon and its transcriptome in a sex-dependent manner
Source: Sci Rep. 2020 Sep 30;10:16160. doi: 10.1038/s41598-020-73166-1 (PMC7527340; doi:10.1038/s41598-020-73166-1)
Supplement: Supplementary file 1 — Supplementary information. [file 41598_2020_73166_MOESM1_ESM.pdf]

Supplementary information for

High-fat diet impacts the colon and its transcriptome in a sex-dependent manner that is modifiable by estrogens

Linnea Hases[1]¶, Amena Archer¶, Rajitha Indukuri, Madeleine Birgersson, Christina Savva, Marion Korach-André, Cecilia Williams

Corresponding author: Cecilia Williams
Email: cecilia.williams@scilifelab.se

This PDF file includes:

Supplementary Fig. 1. Sex-differences in fat distribution and effects of estrogenic treatments in females.
Supplementary Table 1. Primer sequences.
Supplementary Table 2. Genes involved in the pathways regulated by HFD in both females and males.

Supplementary Figures

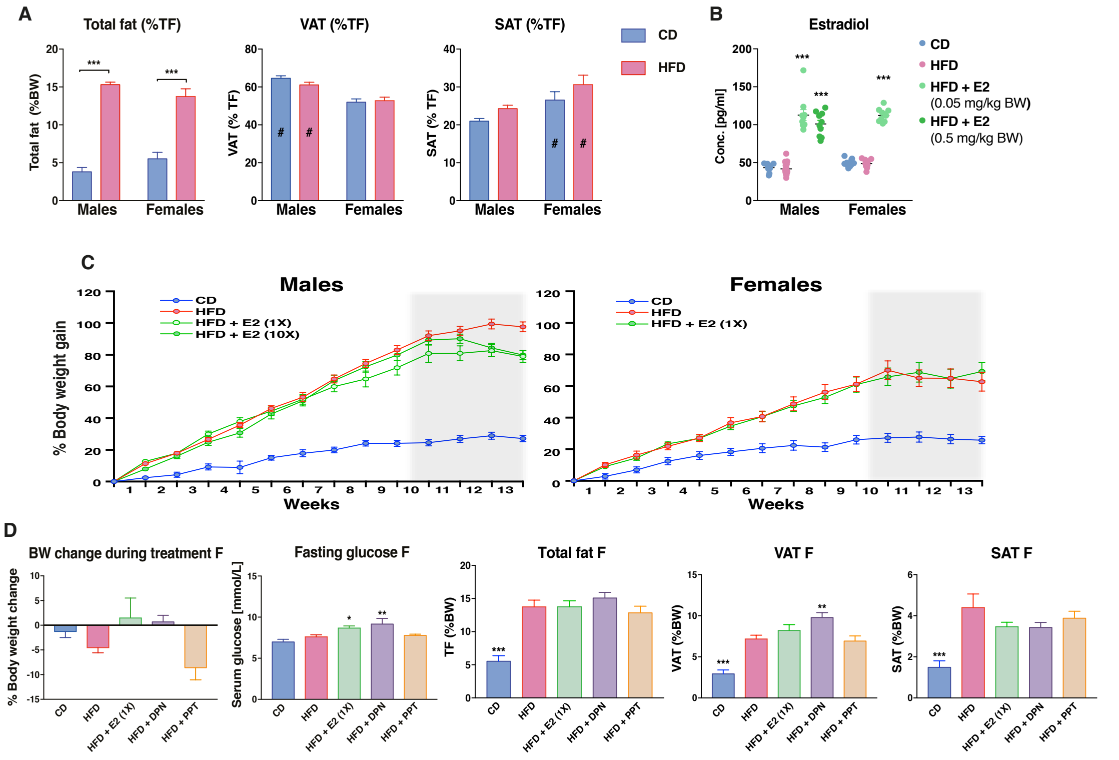

Supplementary Fig. 1. Sex-differences in fat distribution and effects of estrogenic treatments in females. (A) Total fat, VAT and SAT reported to total fat for males and females fed a CD or HFD. **(B)** Serum estradiol levels for females and males on CD, HFD and treated with estrogen (E2). **(C)** BW curves for males and females fed CD or HFD for a 13-weeks period and treated with E2 or vehicle for the last 3 weeks (highlighted in grey). **(D)** BW change during treatment, fasting glucose levels, total fat, VAT and SAT for females under a CD or HFD and treated with vehicle or different estrogenic ligands. One-way and two-way ANOVA with uncorrected Fisher's LSD test, all conditions were compared to HFD vehicle and *p<0.05, **p<0.01, ***p<0.001. # Indicate sex-differences.

Supplementary Tables

Supplementary Table 1. Primer sequences.

Gene	Forward primer (5' - 3')	Reverse primer (5' - 3')
**Genotyping primers**		
Villin Cre	CAA GCC TGG CTC GAC GGC C	
Cre -50 antisense	ATC GAC CGG TAA TGC AGG CA	
ISP (loxP site)	TAG GGT ATG TTA TGT CAT GA	
BIASP (loxP site)	GTG GAT GCC TAT GAT CAC TGT GGA	
**qPCR primers**		
mAnxa1	AAGGTGGTCCTGGGTCAGC	TGAGCATTGGTCCTCTTGGT
mArg1	CTCCAAGCCAAAGTCCTTAGAG	GGAGCTGTCATTAGGGACATCA
mArntl(Bmal1)	ACATAGGACACCTCGCAGAA	AACCATCGACTTCGTAGCGT
mB-actin	GGCTGTATTCCCCTCCATCG	CCAGTTGGTAACAATGCCATGT
mCxcl5	GTTCCATCTCGCCATTCATGC	GCGGCTATGACTGAGGAAGG
mEef2	CATCCTTGCGAGTGTCAGTGA	TGTCAGTCATCGCCCATGTG
mGsto1	CGAACCTAAGGGAAGCGTTGGA	TTCCAGTCGCTGAAACCAAGGC
mNos2	CAGCTGGGVTGTACAAACCTT	CATTGGAAGTGAAGCGTTTCG
mNpas2	ACGCAGATGTTCGAGTGGAAA	CGCCCATGTCAAGTGCATT
mStk11	GGGCAACCTGCTACTCACC	GTCATCCACAGCGAAAGGGT
mTbp	GCAGCAAATCGCTTGGGATTA	ACCGTGAATCTTGGCTGTAAAC

Supplementary Table 2. Genes involved in the pathways regulated by HFD in both females and males.

HFD regulated genes in males			HFD regulated genes in females		
**Angiogenesis**	Ephb2	Egf	**Cell adhesion**	Cd44	Flrt2
	Rapgef3	Fgfr1		Epha1	Flrt3
	Arhgap22	Fn1		Fat2	Gpnmb
	Angpt2	Hand2		Perp	Itgae
	Cspg4	Ncl		Ajuba	Itgam
	Col4a1	Tspan12		Celsr2	Lgals3bp
	Col4a2	Thy1		Cadm4	Muc4
	Col8a1	Tnfrsf12a		Clca2	Nectin1
	Col18a1			Col6a1	Nectin4
**G1/S transition of mitotic cell cycle**	E2f5	Ccnd1		Dsc1	Pkp1
	Ranbp1	Ccnd2		Dsc3	Sdk1
	Anp32b	Inhba		Dsg1b	Tenm2
	Camk2b	Id4		Dsg3	Thbs2
**Positive regulation of cell proliferation**	Dot1l	Fgf7		Efs	Trip6
	Ets1	Fgfr1	**Lipid metabolic process**	Dhcr24	Neu3
	Gli2	Fn1		Acsbg1	Pla2g5
	Rac2	Hmgn5		Alox12	Pld2
	Anp32b	Id4		Alox12b	Psapl1
	Chp2	Klb		Aspg	Sptlc3
	Carm1	Mab21l2		Cers3	Sptssb
	Col18a1	Myc		Dgat2	Smpdl3b
	Ccnd1	Myocd		Elovl4	Scd1
	Ccnd2	Prdx3		Fabp5	Scd3
	Edn1	Plac8		Far2	Them5
	Egf	Sdcbp		Gdpd2	Thrsp
	Fabp4	Tnc		Lipm	Mgll
**DNA replication**	Chaf1a	Mcm4		Lpcat4	
	Chaf1b	Mcm5	**Cell migration**	Epha1	Igf1
	Dtl	Mcm7		Tiam1	Myoc
	Mcm2	Nfic		Alox12	Pld2
	Mcm3	Rpa3		Col1a1	Sema3d
**Cell cycle**	Ddx11	Incenp		Cpeb1	Sema3f
	E2f7	Lmln		Gpnmb	Snai2
	E2f8	Mdc1		Hbegf	Trip6
	Fbxl7	Mcm2	**Steroid hormone mediated signaling pathway**	Ar	Nr4a1
	Mad1l1	Mcm3		Bmp7	Pgr
	Trnp1	Mcm4		Esr1	Rarg
	Wee1	Mcm5		Nr1d2	
	Cep250	Mcm7	**Negative regulation of cell proliferation**	Dhcr24	Hmox1
	Chaf1a	Rcc2		Bcl6	Igf1
	Chaf1b	Stk10		H19	Igfbp3
	Cit	Stk11		Sox9	Ifit3
	Clspn	Ttc28		Ar	Rarg
	Ccnd1	Uhrf1		Bmp7	Robo1

	Ccnd2	
**Positive regulation of cell migration**	Ets1	Igf1r
	Actn4	Itga5
	Cdh13	Itgb3
	Col18a1	Pik3cd
	Edn1	Sema4c
	Fn1	Sdcbp
**Glucose homeostasis**	Ero1lb	Kcnb1
	Gpr21	Rph3al
	Cacna1e	Stk11
	Gk	Sirt6
	Pck1	
**Positive regulation of T cell proliferation**	Sash3	Tfrc
	Il1b	Tnfrsf13c
	Ptprc	Vcam1
**Immune response**	Tgtp1	H2-DMb2
	Ccl6	Igf1r
	Ccr6	Il1b
	C7	Lst1
	H2-Oa	Lax1
	H2-Ob	Mcpt4
	H2-Aa	Plscr1
**Cell adhesion**	Aoc3	Lmln
	Boc	Mfap4
	Cdh13	Pip5k1c
	Cercam	Ptpru
	Col8a1	Sell
	Col15a1	Spon2
	Col18a1	Tnc
	Fn1	Thy1
	Itga5	Tnfrsf12a
	Itgb3	Vcam1
	Lamc1	
**Response to hypoxia**	Bnip3	Edn1
	Ets1	Myocd
	Ascl2	Nos1
	Actn4	Sod3
	Angpt2	Vcam1
	Cygb	
	Cpeb1	Scin
	Foxa3	Sfrp2
	Gja1	Zfp503
**Positive regulation of cell proliferation**	Epha1	Grem1
	Isl1	Gas1
	Sox9	Hbegf
	Tiam1	Igf1
	Tbx3	Lifr
	Atf3	Mzb1
	Aldh3a1	Nccrp1
	Areg	Osr2
	Ar	Pgr
	Alox12	Rarg
	Edn2	Sfrp2
**Immune system process**	Oasl1	Ifit3
	Bcl6	Il1f9
	Prdm1	Ltf
	S100a8	Lilrb4a
	Gbp5	Lbp
	Hp	Rnf125
	Iigp1	Slpi
	Irf7	Smpdl3b
	Ifit1	
**Positive regulation of apoptotic process**	Bcl6	Igfbp3
	Bok	Il18
	Acvr1c	Nr4a1
	Aldh1a3	Phlda3
	Alox12	Rarg
	Bmp7	Scin
	Dusp6	Sfrp2
	Hmox1	
**Negative regulation of canonical Wnt signaling pathway**	Isl1	Sfrp2
	Sox9	Snai2
	Grem1	Wnt4
	Nkd2	
**Positive regulation of MAPK cascade**	Dnajc27	Igf2
	Ar	Igfbp3
	Alox12b	Igfbp4
	Igf1	
**Angiogenesis**	Epha1	Hbegf
	Meis1	Il18
	Adam8	Ramp2
	Ecm1	Tnfaip2
	Grem1	Vav3
	Hmox1	
